# High rate lithium-sulfur battery enabled by sandwiched single ion conducting polymer electrolyte

**DOI:** 10.1038/srep22048

**Published:** 2016-02-22

**Authors:** Yubao Sun, Gai Li, Yuanchu Lai, Danli Zeng, Hansong Cheng

**Affiliations:** 1 Sustainable Energy Laboratory, Faculty of Materials Science and Chemistry, China University of Geosciences (Wuhan) 388 Lumo RD, Wuhan 430074, China

## Abstract

Lithium-sulfur batteries are highly promising for electric energy storage with high energy density, abundant resources and low cost. However, the battery technologies have often suffered from a short cycle life and poor rate stability arising from the well-known “polysulfide shuttle” effect. Here, we report a novel cell design by sandwiching a *sp*^3^ boron based single ion conducting polymer electrolyte film between two carbon films to fabricate a composite separator for lithium-sulfur batteries. The dense negative charges uniformly distributed in the electrolyte membrane inherently prohibit transport of polysulfide anions formed in the cathode inside the polymer matrix and effectively blocks polysulfide shuttling. A battery assembled with the composite separator exhibits a remarkably long cycle life at high charge/discharge rates.

Sulfur is recognized as one of the most promising cathodic materials for the next generation of lithium ion batteries due to its high earth abundance and intrinsically low cost. Lithium sulfur batteries are capable of offering an extraordinarily high discharge capacity of 1675 mAh•g^−1^ and a high power density up to 2600 Wh•kg^−1 ^1,2,3 and thus well suited for a wide range of applications. Nevertheless, efforts to commercialize Li-S battery technologies have been severely hampered by the technical challenge to stabilize the cathode in an ether electrolyte^4^,^5^,^6^ during battery operation largely due to the well-known polysulfide shuttling effect^7^,^8^,^9^,^10^,^11^,^12^,^13^. Containment of sulfur in a conductive framework has been the primary objective of research for enhancing the battery performance. One of the main strategies is to encapsulate sulfur to improve the interfacial contact and to limit the mobility of polysulfides. The techniques include impregnating sulfur in porous carbon materials^15^,^16^,^17^,^18^,^19^,^20^,^21^,^22^,^23^,^24^, coating with graphene fibers^25^,^26^, wrapping with conductive polymers^27^,^28^,^29^,^30^, and binding polysulfides with nitrogen doped carbons^31^,^32^. Although considerable improvement of battery longevity has been achieved, the effectiveness on sulfur containment is still limited and the methods for cathode fabrication are hardly scalable for commercial scale production. Attempts have been made to employ functionalized polymeric binders^33^,^34^,^35^,^36^,^37^ or metal oxides with designated sites for absorption^38^,^39^,^40^ to stabilize the cathode. However, the nonconductive nature of these materials results in relatively poor rate performance. In essence, none of the techniques that have been developed to date to contain sulfur offers a simple but effective solution to the polysulfide shuttling problem. Instead, the majority of these techniques are to increase the electronic contact and/or anchor polysulfides in the cathode. Indeed, it is a technically formidable challenge to develop an effective method to achieve complete containment of polysulfides in the cathode.

A lithium ion exchange membrane is a single ion conductor serving as an electrolyte in batteries^41^,^42^,^43^,^44^. The anions are anchored in the polymer framework and thus immobile, while lithium ions are well-separated from the counter-ions with high mobility. The dense negative charges distributed in the electrolyte membrane thus inherently prohibit transport of polysulfide anions formed in the cathode inside the polymer matrix due to the strong electrostatic repulsion. Therefore, with the installation of an appropriate single ion electrolyte membrane in a Li-S battery device, it becomes possible to shut down polysulfide shuttling between electrodes. Here, we demonstrate that a highly conductive novel single ion electrolyte composite membrane is capable of enabling excellent performance of a Li-S battery at high C-rates (Fig. 1).

The composite membrane was made from a *sp*^3^ boron-based single ion polymer (PDTAB) sandwiched between two carbon films on top of a Celgard film. Unlike lithiated Nafion^45^,^46^,^47^, PDTAB was specifically designed to block polysulfide species while maintaining high ionic conductivity in non-aqueous solutions. One carbon black film in contact with sulfur serves as a reservoir to store soluble polysulfides and another in contact with the Celgard film acts as a bridge to reduce interfacial resistance. A Li-S battery assembled with this composite separator exhibits outstanding performance at high charge/discharge rates with remarkably high capacity retention and a long cycle life. The methods used for material synthesis and device fabrication can be easily scaled to allow low cost, mass production of Li-S batteries.

## Results

### Synthesis and characterization of the single ion polymer electrolyte (PDTAB) film

**Figure 2:**
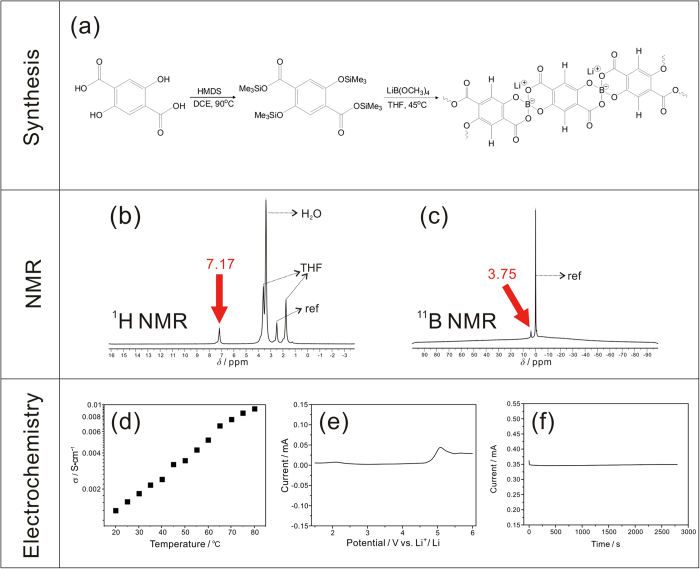
Synthesis and characterization of the PDTAB.

The synthetic route, structural characterization and electrochemical properties are shown in Fig. 2. Silylated 2,5-dihydroxyterephthalic acid was reacted with stoichiometric tetramethanolatoborate to obtain a *sp*^3^ borate polymer (Fig. 2(a)). Its chemical structure was analyzed with ^1^H NMR (Fig. 2(b)) and ^11^B NMR (Fig. 2(c)). As expected, a single peak appears at 7.17 ppm corresponding to the hydrogen on the benzene ring from the target product. The single peak at 3.75 ppm of the ^11^B NMR spectrum indicates the formation of *sp*^3^ hybrid boron^48^,^49^. The gel permeation chromatograph (GPC) analysis indicates that the weight average molecular weight of PDTAB and the polydispersity index are 6,766 and 1.12, respectively. The PDTAB electrolyte film is the mixture of PDTAB ionomer and PVDF-HFP by a solution cast method. The ionic conductivity of the PDTAB electrolyte film is 1.59 mS•cm^−1^ at room temperature (Fig. 2(d)). It is electrochemically stable in the range of 1–4.5 V (vs. Li^+^/Li) (Fig. 2(e)). The chronoamperametry test (Fig. 2(f)) shows that the current response to a constant voltage step remains essentially invariant for at least up to 45 minutes, indicating that lithium ions are responsible for the overall charge transport at a constant rate. The lithium ion transference number was derived from the ratio of the steady current and the initial current^50^ and found to be 0.95, sufficiently close to unity. The results suggest that the PDTAB membrane is indeed a typical single ion polymer electrolyte with high ionic conductivity and strong electrochemical stability.

### The role of the sandwiched composite separator

**Figure 3:**
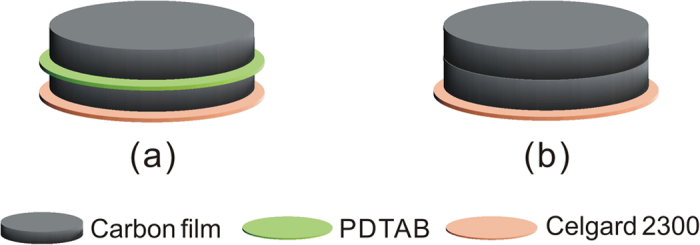
Configurations of the composite separators (**a**) from bottom: Celgard film, carbon film|single ion polymer film|carbon film; (**b**) from bottom: Celgard film, carbon film|carbon film.

To highlight the role of the single ion electrolyte membrane in blocking the polysulfide species, two configurations of the composite membranes were considered as schematically depicted in Fig. 3. Figure 3(a) illustrates a composite membrane made of a PDTAB film sandwiched between two carbon films with a Celgard film at the bottom. The composite membrane was placed between a sulfur cathode and a lithium metal foil anode to make a battery cell, similar to how a conventional lithium-sulfur battery is assembled^51^. For comparison, a blank experiment using a cell without the PDTAB film was also conducted (Fig. 3(b)).

**Figure 4:**
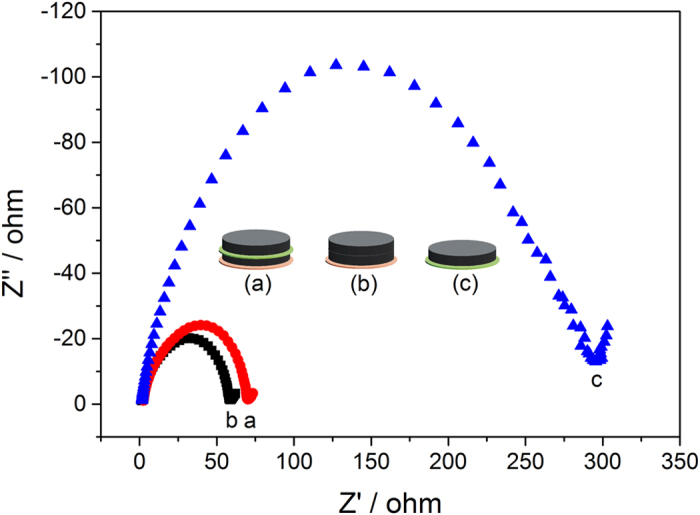
The EIS spectra of the three different cell configurations (**a**) from bottom: Celgard film, carbon film|single ion polymer film|carbon film; (**b**) from bottom: Celgard film, carbon film|carbon film; (**c**) from bottom: Single ion polymer film|carbon film.

The necessity of the Celgard film and the carbon film close to the anode is evidenced from the comparison of the electrochemical impedance spectra shown in Fig. 4. The interfacial resistance of the cell with the composite membrane (Fig. 4(a)) was found to be 70 Ω. Upon removal of the PDTAB film, the interfacial resistance decreases slightly to 59 Ω due to the shorter transport distance of the ions. Furthermore, if both the carbon and the Celgard films are removed, the interfacial resistance rises up sharply to 296 Ω. Clearly, carbon and Celgard film are capable of reducing the interfacial resistance by turning the poor interfacial contact between the dense PDTAB film and the Li-foil into a soft contact between the anode and the porous film saturated with a liquid electrolyte (1 M lithium trifluoromethanesulfonimide (LiTFSI) in the mixed solvent of dimethoxyethane (DME) and 1,3-dioxolane (DOL) (1:1, v:v)).

### Battery performance

**Figure 5:**
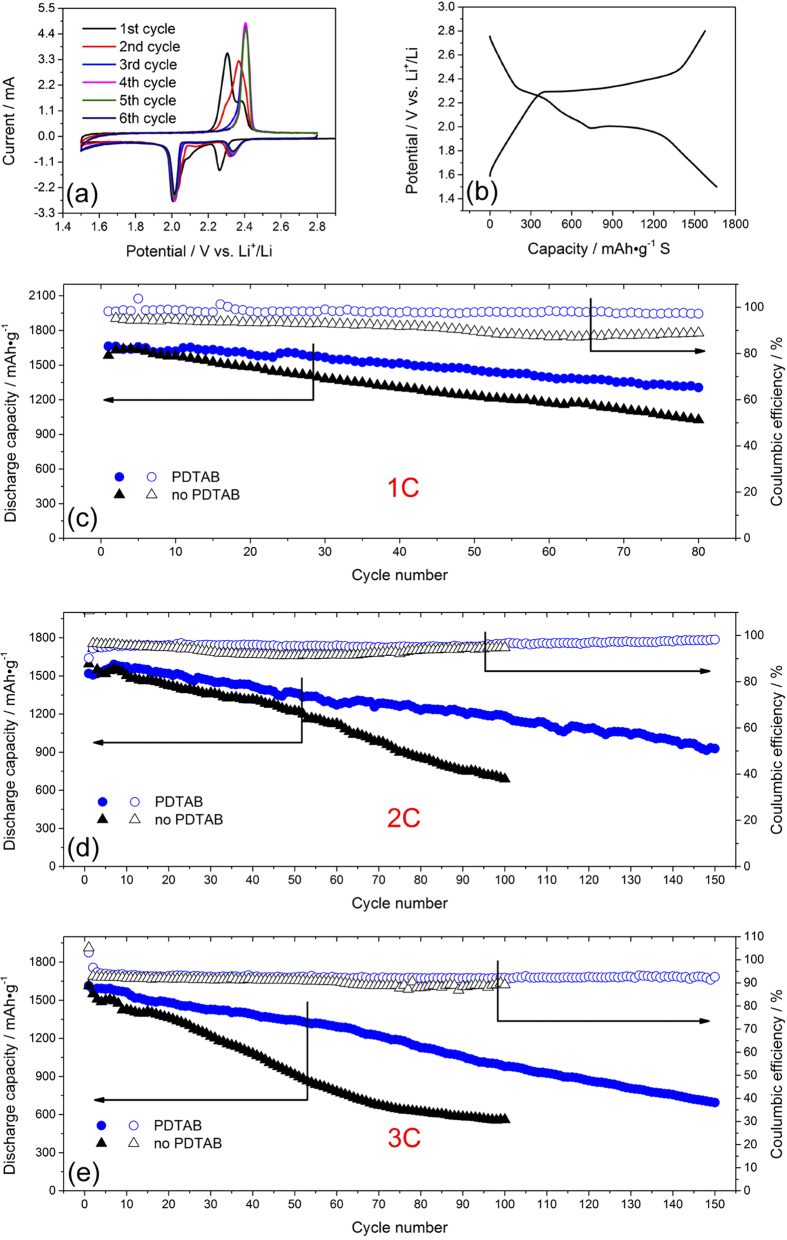
Electrochemical measurement of lithium sulfur batteries (**a**) cyclic voltammetries of the cell (Fig. 3(a)) at a scan rate of 1mV/s; (**b**) the 1^st^ discharge/charge curves of the cell (Fig. 3(a)) at 1 C rate; (**c**) cycle performance of the cells (Fig. 3) at 1 C rate; (**d**) cycle performance of the cells (Fig. 3) at 2 C rate; (**e**) cycle performance of the cells (Fig. 3) at 3 C rate.

Cyclic voltammetry was conducted to study the electrochemical behavior of the assembled lithium sulfur batteries (Fig. 5(a)). In the first cycle, two typical cathodic peaks and two overlapping anodic peaks are clearly observable with the peak potentials of 2.26 V, 2.00 V, 2.30V and 2.38V, respectively, consistent with the reported CV profiles of sulfur^46^. The cathodic peak at 2.26 V corresponds to the formation of soluble long chain polysulfides, which are then electrochemically reduced to an insoluble short chain polysulfide, forming another cathodic peak at 2.0 V. The anodic peak potential lags behind the cathodic peak potential due to the irreversible polarization resistance. In the subsequent cycles, the two anodic peaks gradually merge into one and move to the high potential, indicating that the oxidation process becomes slightly more irreversible. In contrast, the area of the cathodic peak at 2.26 V decreases gradually and the peak potential moves upward, suggesting that the reduction of sulfur to soluble polysulfides becomes more favorable.

Figure 5(b) shows the 1^st^ discharge and charge curves at 1 C rate. In the discharge curve, two typical plateaus, from 2.3 V to 2.0 V and from 2.0 V to 1.5 V, respectively, are clearly visible. The first plateau represents the conversion from sulfur to soluble polysulfides with long chains and the second one reflects the conversion from soluble polysulfides to indissoluble Li_2_S^46^. The observed two plateaus are consistent with the two cathodic peaks found in the cyclic voltammetry curves shown in Fig. 5(a). In the charge curve, there is one plateau, which corresponds to the anodic peak in the cyclic voltammetry curve. The gap between the charge and discharge curves is around 300 mV, close to the peak potential difference between the cathodic peak and the anodic peak. This voltage gap arises from the electric polarization and the electrochemical reaction polarization.

In order to evaluate the battery performance objectively, the specific capacities of sulfur are corrected by deducting the electrochemical double layer capacitance^52^ from the measured capacities. The battery performance of the two configurations of the composite separators at various C-rates is shown in Fig. 5(c–e). Detailed results are summarized in Table 1. As anticipated, the battery with the composite separator containing the single ion polymer electrolyte film is substantially more effective in inhibiting polysulfide shuttling than the cell without the PDTAB film with a much lower decay rate, higher capacity retention and better coulombic efficiency. The cells with PDTAB exhibit an average decay rate of 4.4 mAh•g^−1^ per cycle at 1 C, 4.7 mAh•g^−1^ per cycle at 2 C and 6.2 mAh•g^−1^ per cycle at 3 C. In comparison, for the cells without PDTAB, their discharge capacities deteriorate more rapidly, exhibiting an average decay rate of 7.2 mAh•g^−1^ per cycle at 1 C, 9.1 mAh•g^−1^ per cycle at 2 C and 10.5 mAh•g^−1^ per cycle at 3 C. In particular, the effect of the PDTAB membrane is more pronounced at a high rate. At 2 C, the cell with PDTAB still maintains a capacity of 1185 mAh•g^−1^ after 100 cycles. In comparison, the discharge capacity of the cell without PDTAB declines to only 688 mAh•g^−1^ with the same cycle number. At the 100^th^ cycle with a C-rate of 3 C, the discharge capacity of the cell with PDTAB decreases modestly to 979 mAh•g^−1^, but for the cell without PDTAB the capacity becomes only 561 mAh•g^−1^. Although the interface resistance is slightly reduced upon removal of the PDTAB film as observed in Fig. 4, the corresponding battery performance is worse than that with PDTAB. In fact, the interface resistance does not necessarily correlate directly the cycle performance. In the case of Li-S batteries, the initial interfacial resistance can be low, which gives rise to a high discharge capacity. However, the cycle performance may be remarkably poor due to the shuttling effect of soluble polysulfides. This is precisely the reason that the single ion conducting polymer electrolyte membrane is needed, which prohibits crossover of the polysulfide anions through the membrane via strong electrostatic repulsion. As a consequence, the cell with the PDTAB film exhibits better cycle stability than the cell without the film.

To examine the role of the carbon films, we conducted an experiment by removing the carbon layers from the composite membrane followed by a test of cell cycle performance. The results are shown in Supporting Information. Not surprisingly, the battery performance was found to be far inferior to what was observed with the cell containing the sandwiched composite membrane because the interfacial resistance between the dense PDTAB electrolyte film and the electrodes is very high, which is consistent with the EIS data (Fig. 4). We believe the synergetic effect of the porous carbon films and the dense PDTAB membrane attributes to the better performance of the cell device.

## Discussion

**Figure 6:**
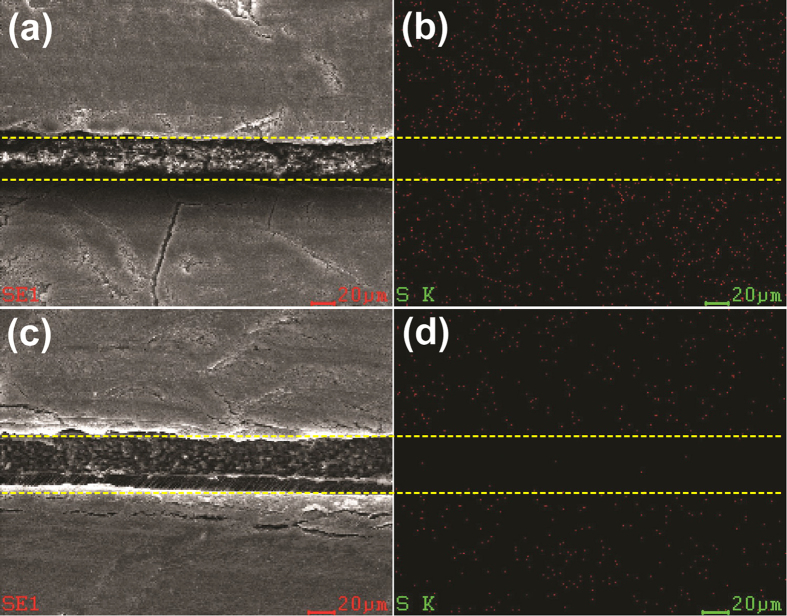
SEM&EDX detection of sulfur distribution in the interlayer (the upper carbon film is close to the anode and the lower carbon film is close to the cathode; the single ion conducting polymer electrolyte film is placed in between) (**a**) The SEM image of the sandwiched separator after 20 discharge/charge cycles; (**b**) sulfur distribution of the sandwiched separator after 20 discharge/charge cycles by EDX; (**c**) The SEM image of the sandwiched interlayer upon wash with DME after 20 discharge/charge cycles; (**d**) sulfur distribution of the sandwiched interlayer upon wash with DME after 20 discharge/charge cycles by EDX.

It is important to note from Fig. 5(c–e) that the initial decay rate for the batteries with the single ion polymer electrolyte is not as steep as what has been observed in other reported Li-S batteries, which usually display a sharp initial capacity decrease^45^. While the carbon film next to the sulfur electrode serves as the first line of defense to store soluble polysulfides, the single ion polymer electrolyte film plays a major role in blocking polysulfide anions via strong electrostatic repulsion. As a result, the polysulfide species are largely controlled in the cathode side at the very beginning. Nevertheless, the discharge curves for the batteries with the single ion polymer electrolyte in Fig. 5 still exhibit steady decline of capacity as the number of cycle increases, which suggests that cathode degradation still occurs. To understand the peculiar battery performance, we conducted *ex-situ* SEM and DEX (Energy Dispersive X-Ray Spectroscopy) analyses on the cell in a charged state after 20 cycles at 1 C to examine the sulfur distribution in the composite membrane. The cell was opened in a glove box. Subsequently, the sandwiched layer was peeled off for the SEM and EDX analyses. The images of the cross section are shown in Fig. 6. Three distinct layers are clearly seen in Fig. 6(a) with the single ion conducting polymer electrolyte membrane in between two carbon films. The elemental sulfur represented by the red dots is clearly visible on both sides of the carbon films as shown in Fig. 6(b). In comparison, very little presence of sulfur is found in the PDTAB layer; the few spotty red dots observed in the PDTAB layer are deemed from the sulfur carriers in the carbon films as a result of surface cutting for film analysis. Upon scratch between the blade and the carbon films, some sulfur species may be left on the PDTAB film. The sources of sulfur are identified from the sulfur electrode and the liquid electrolyte (LiTFSI) which contains sulfur in its molecular structure and is used in the battery cell to enhance device performance. To differentiate the sulfide species from the TFSI anions, the composite interlayer was subsequently immersed in DME for 24 hours and then washed with DME three times to remove the residual LiTFSI. The material was then subject to SEM and EDX analysis with the images shown in Fig. 6(c,d). An essentially blank PDTAB layer is readily seen in Fig. 6(d), similar to what was observed in Fig. 6(b). Surprisingly, there is still a plenty of sulfur found in both carbon films even upon the removal of LiTFSI, indicating that leakage of polysulfides from the cathode to the anode occurs by the periphery path.

To further confirm the critical role of PDTAB in blocking polysulfide shuttling, we designed an experiment using U-shaped glass electrolysis cells with sulfur as an electrode and a lithium foil as the counter-electrode. Subsequently, a Celgard film, a sandwiched Celgard film and a sandwiched PDTAB film were respectively placed in between the electrodes. The devices were filled with a commercial electrolyte (LiTFSI in DOL and DME) and placed in a glove box followed by galvanostatic discharge for over 72 hours. Details of the testing results are shown in Supporting Information. As expected, the anolyte became yellow shortly after 12 hours upon the electrolysis with the Celgard film, indicating that sulfide species were generated upon the electrochemical reduction of sulfur and diffused across the film from the catholyte to the anolyte. For the sandwiched Celgard film the color of the anolyte became slightly yellow after 48 hours of the electrolysis, and gradually turned to bright yellow after 72 hours upon the electrolysis. In contrast, for the sandwiched PDTAB film the color of the anolyte remained essentially unchanged even after 72 hours upon the electrolysis. Details of the testing results are shown in Supporting Information. Therefore, two important conclusions can thus be drawn from the above experimental observations:

1. The single ion electrolyte membrane PDTAB is indeed capable of blocking anionic species including polysulfides and TFSI;
2. Leakage of sulfide species takes place likely through the periphery in the coin cell facilitated by the liquid electrolyte LiTFSI.

A possible mechanism is that the polysulfide species initially are accumulated in the carbon film in contact with the sulfur electrode and then diffuse through the periphery to the anodic side driven by the concentration gradient. The leaking effect may account for the steady decay of discharge capacity observed in Fig. 5(c–e). Clearly, a capability to strictly separate the cathode from the anode by a single ion conducting polymer electrolyte is essential to avoid the leakage for further enhancement of the battery performance.

## Methods

### Synthesis and preparation of single ion polymer electrolyte film

Initially, 2,5-dihydroxyterephthalic acid was silylated by hexamethyldisilazane. Upon purification, 1.276 g (2.5 mmol) of silylated product was reacted with 0.3547 g (2.5 mmol) of tetramethanolatoborate in anhydrous tetrahydrofuran at 45 ^o^C for three days for condensation. A white precipitate was filtrated, washed with anhydrous tetrahydrofuran several times and dried at 60 ^o^C under vacuum for 2 days. The product was named PDTAB. 0.6 g of PVDF-HFP and 0.6 g of PDTAB were added to 10 mL of an anhydrous N-methylpyrrolidinone (NMP) solvent under stirring to obtain a homogeneous solution. Subsequently, the solution was cast onto a flat glass substrate and dried at 50 ^o^C in open air. A piece of ~25 μm thick composite film was formed upon solvent removal. The as-prepared single ion conducting polymer electrolyte film was dried at 80 ^o^C under high vacuum for several days for further use.

### NMR characterization

Both ^1^H NMR and ^11^B NMR were taken on a 400 MHz instrument (AVANCE Ш HD 400 MHz, Swiss BRUKER) in deuterated dimethyl sulfoxide (DMSO-*d*_6_) at room temperature. For the ^11^B NMR test, BF_3_•Et_2_O was sealed in a glass capillary tube as the internal reference.

### Weight average molecular weight

Molecular weight and polydispersity index (PDI) were measured with gel permeation chromatography (Waters 515 HPLC Pump, Waters 2707 Autosampler, Waters 2414 Refractive Index Detector).

### Electrochemical characterization of the single ion conducting polymer electrolyte

(a) Ionic conductivity. The single ion conducting polymer electrolyte film was cut into a disk soaked with a solution of ethylene carbonate and propylene carbonate at the volume ratio of 1:1. The film was then inserted into the stainless steel blocking electrodes. Electrochemical impedance spectroscopy (EIS) was set up from 1 Hz to 1 MHz with the AC voltage amplitude of 10 mV at the open circuit voltage. (b) Electrochemical stability. Similar to the ionic conductivity test, a piece of lithium metal foil was selected to serve as the counter electrode as well as a reference electrode. Linear scanning voltammetry was conducted from 0.8 V to 6.0 V at 1 mV/s. (c) Ion transference number. The transference number was measured using the blocking electrode with the single ion conducting polymer electrolyte film sandwiched between two pieces of lithium metal foils. Current was recorded at a fixed potential of 10 mV.

### Coil cell assembly and test

Black pearl was mixed with 30 wt% PTFE to prepare a carbon film with a thickness of around 180 μm. One piece of PDTAB film was sandwiched between two pieces of carbon films upon oil press. All films have the same diameter of 18 mm. This composite film was dried at 100 ^o^C under vacuum for one day. The cathode film was prepared via a solution cast method. Pure sulfur, acetylene carbon, and a PVDF binder with the mass ratios of 70 wt%, 20 wt% and 10 wt%, respectively, are mixed in a NMP solvent under stirring for 6 hours. Subsequently, the mixture was coated on a smooth aluminum substrate using a blade. Upon solvent removal at 50 ^o^C in an oven, it was punched to several small rounds with a diameter of 15 mm. The sulfur loading on each disc is about 1.5 mg. Before assembling coin cells, these cathode discs were dried at 50 ^o^C under vacuum for at least one day. The liquid electrolyte used in the cells contains 1.0 M LiTFSI dissolved in 1,2-dimethoxyethane (DME) and 1,3-dioxolane (DOL) at 1:1 volume ratio. The amount of liquid electrolyte in each cell is around 50 μL. CR2025 coin cells were assembled in a glove box. Batteries were measured on an Arbin tester with cutoff potentials of 1.5 V and 3.0 V at various rates.

### Morphorlogy and sulfur distribution analysis on the composite interlayer

The coin cells after test were transferred into a glove box. They were opened with a pair of pliers. The sandwiched PDTAB film was peeled off and cut into strips for analysis. One piece of the strips was immersed in anhydrous 1,2-dimethoxyethane (DME) to leach out the liquid electrolyte. Subsequently, they were washed several times with anhydrous 1,2-dimethoxyethane followed by vacuum dry.

## Additional Information

**How to cite this article**: Sun, Y. *et al.* High rate lithium-sulfur battery enabled by sandwiched single ion conducting polymer electrolyte. *Sci. Rep.* **6**, 22048; doi: 10.1038/srep22048 (2016).

## Supplementary Material

Supplementary Information

## Figures and Tables

**Figure 1:**
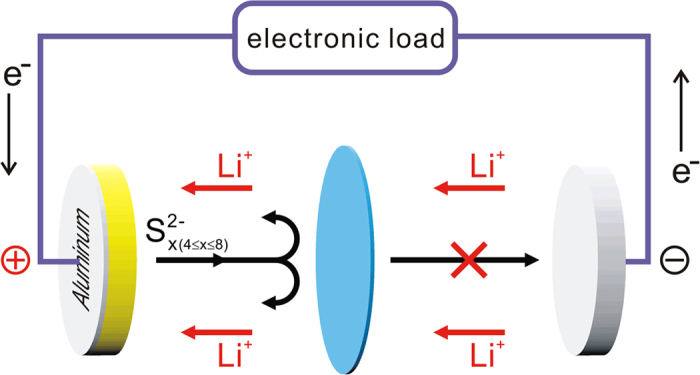
Scheme of the composite separator.

**Table 1:** Battery performance comparisons between cells (a) and (b) in Fig. 3.

	1^st^discharge capacity/mAh•g^−1^ S	Final discharge capacity/mAh•g^−1^ S	Average decay rate/mAh•g^−1^ S	Average coulombic efficiency/%	
1C	PDTAB (80 cycles)	1662	1306	4.4	98
	Blank (80 cycles)	1584	1026	7.2	91
2C	PDTAB (150 cycles)	1519	928	4.7	96
	Blank (100 cycles)	1594	688	9.1	94
3C	PDTAB (150 cycles)	1614	695	6.2	92
	Blank (100 cycles)	1614	561	10.5	90
