# Supplementary material for: High rate lithium-sulfur battery enabled by sandwiched single ion conducting polymer electrolyte
Source: Sci Rep. 2016 Feb 22;6:22048. doi: 10.1038/srep22048 (PMC4761998; doi:10.1038/srep22048)
Supplement: Supplementary Information [file srep22048-s1.doc]

Supporting Information

**High rate lithium-sulfur battery enabled by sandwiched single ion conducting polymer electrolyte**

Yubao Sun^1^, Gai Li^1^, Yuanchu Lai^1^, Danli Zeng^1^ & Hansong Cheng^1^

^1^Sustainable Energy Laboratory, Faculty of Materials Science and Chemistry, China University of Geosciences (Wuhan) 388 Lumo RD, Wuhan 430074, China. Correspondence and requests for materials should be addressed to Y.S. (email: sunyubao@gmail.com) or to H.C. (email: chghs2@gmail.com).

**Leakage measurement.** To confirm the critical role of PDTAB in blocking polysulfide shuttling, we designed a qualitative experiment using U-shaped glass electrolysis cells with sulfur as an electrode and a lithium foil as the counter-electrode (Fig. S1, S2 and S3). The separators placed in between are Celgard film, sandwiched Celgard film and sandwiched PDTAB film respectively. The devices were filled with a commercial electrolyte (LiTFSI in DOL and DME) and placed in a glove box followed by galvanostatic discharge for over 72 hours. As expected, with the Celgard film, the anolyte became yellow shortly after 12 hours upon the electrolysis, indicating that sulfide species were generated upon the electrochemical reduction of sulfur and diffused across the film from the catholyte to the anolyte. For the sandwiched Celgard film the color of the anolyte became slightly yellow after 48 hours upon the electrolysis, and gradually changed to bright yellow after 72 hours upon the electrolysis. In contrast, for the sandwiched PDTAB film the color of the anolyte remained essentially unchanged even after 72 hours upon the electrolysis.


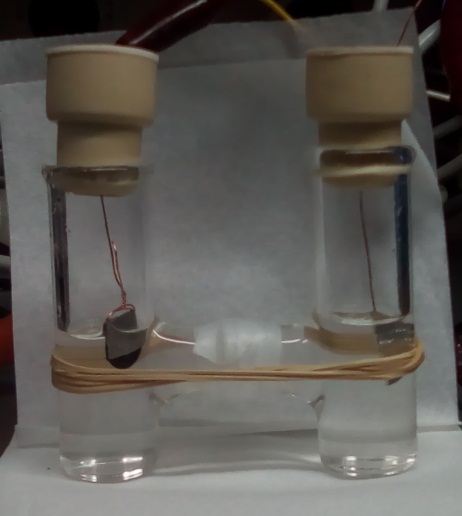

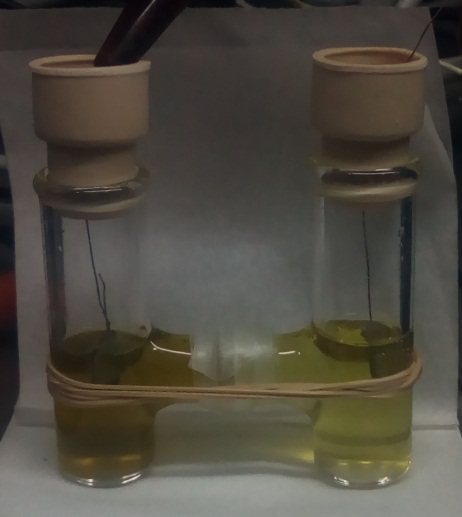


0 12 hours

Figure S1. The electrolysis cell with a Celgard film as the separator.


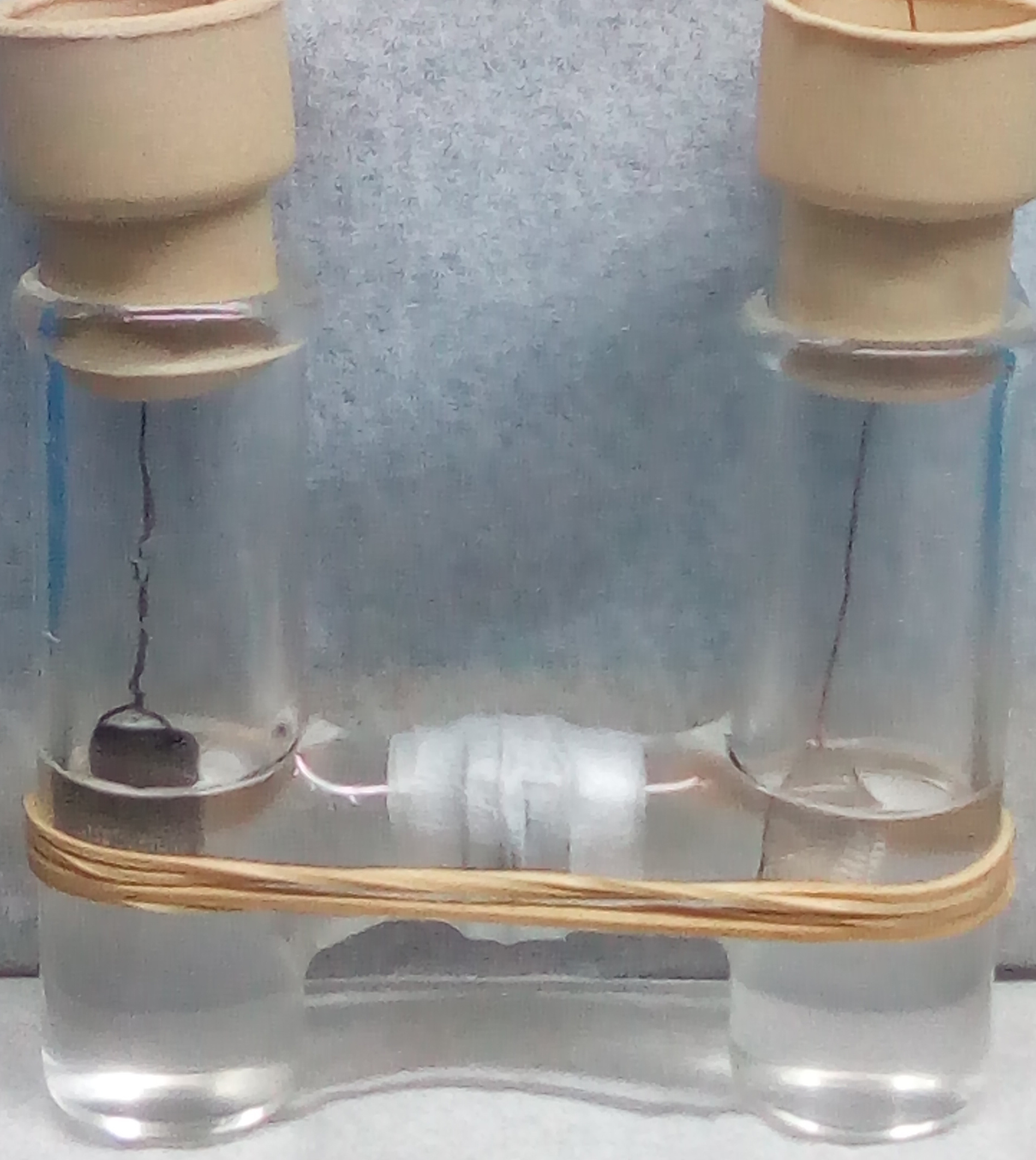

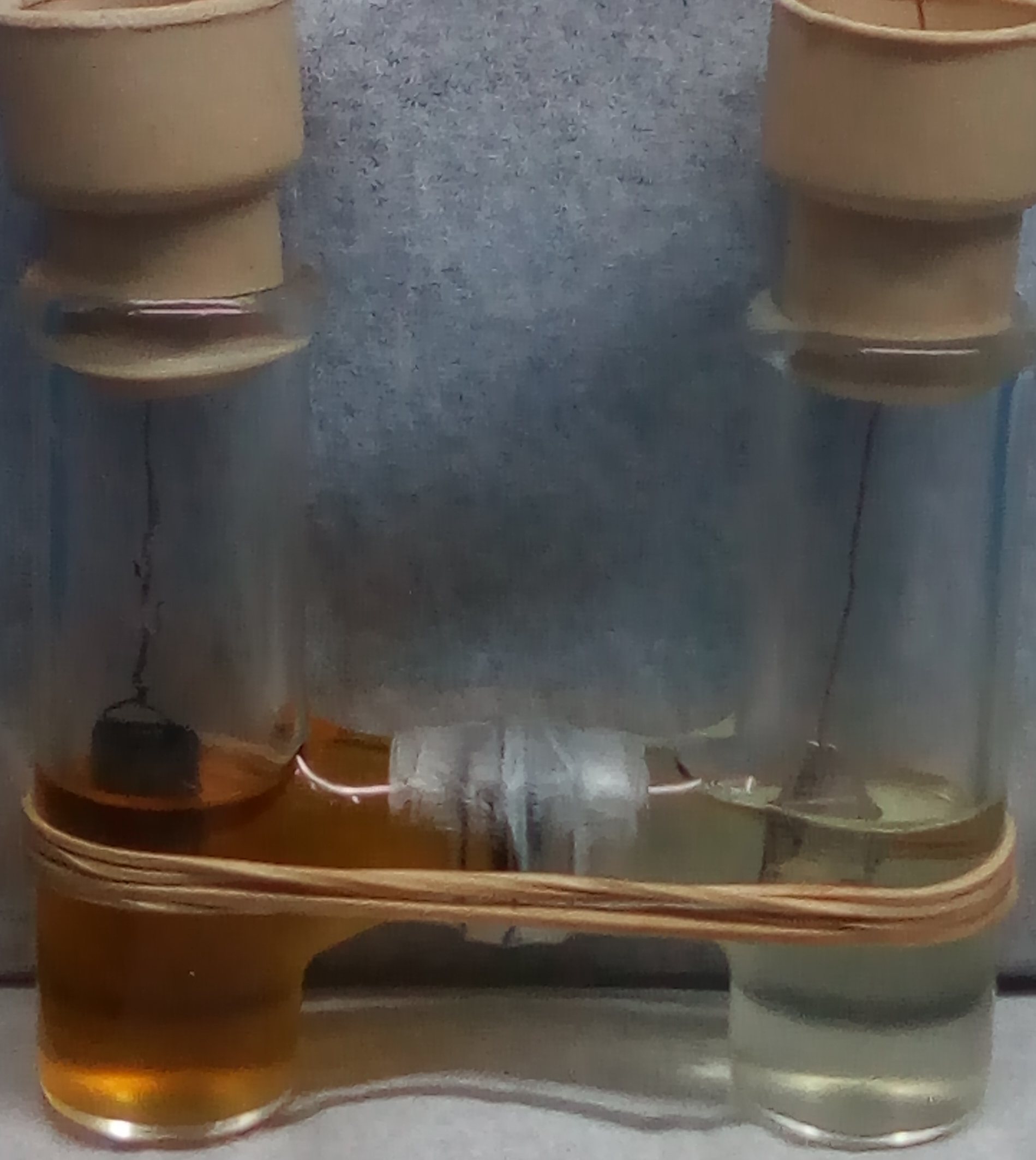

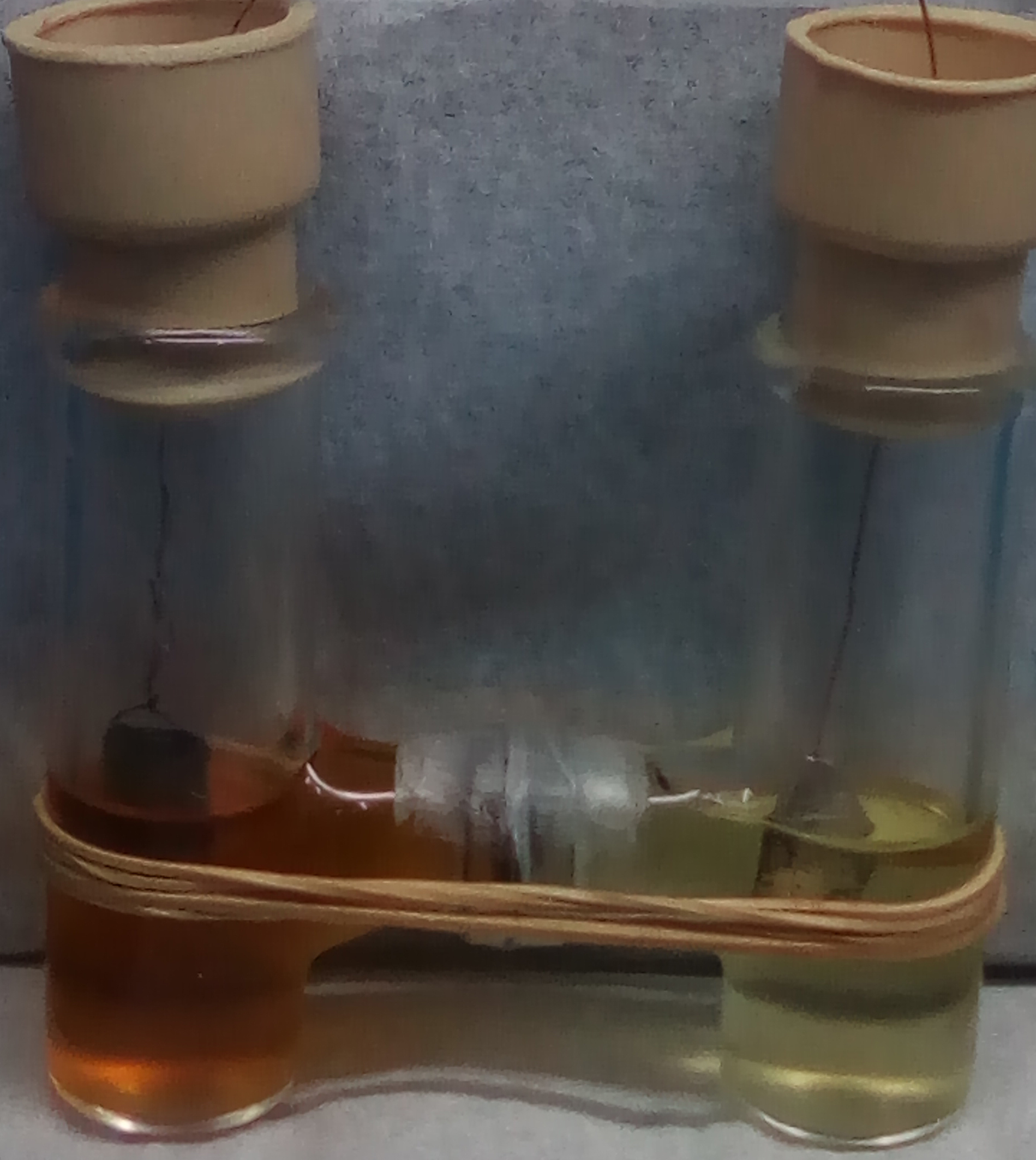


0 48 hours 72 hours
Figure S2. The electrolysis cell with the sandwiched Celgard film as the separator


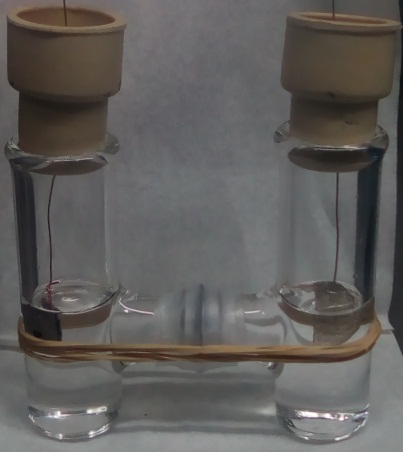

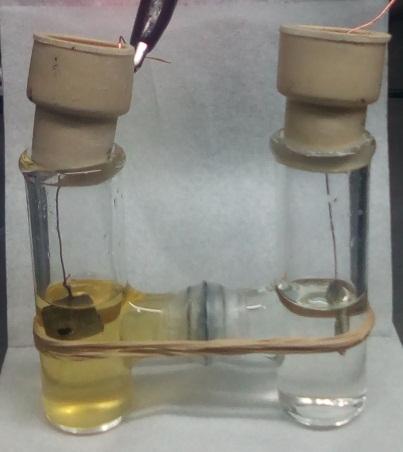

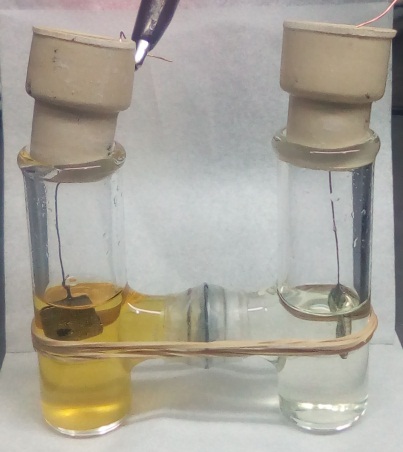


0 48 hours 72 hours
Figure S3. The electrolysis cell with the sandwiched PDTAB as the separator.

**Blank battery test with PDTAB film only.**


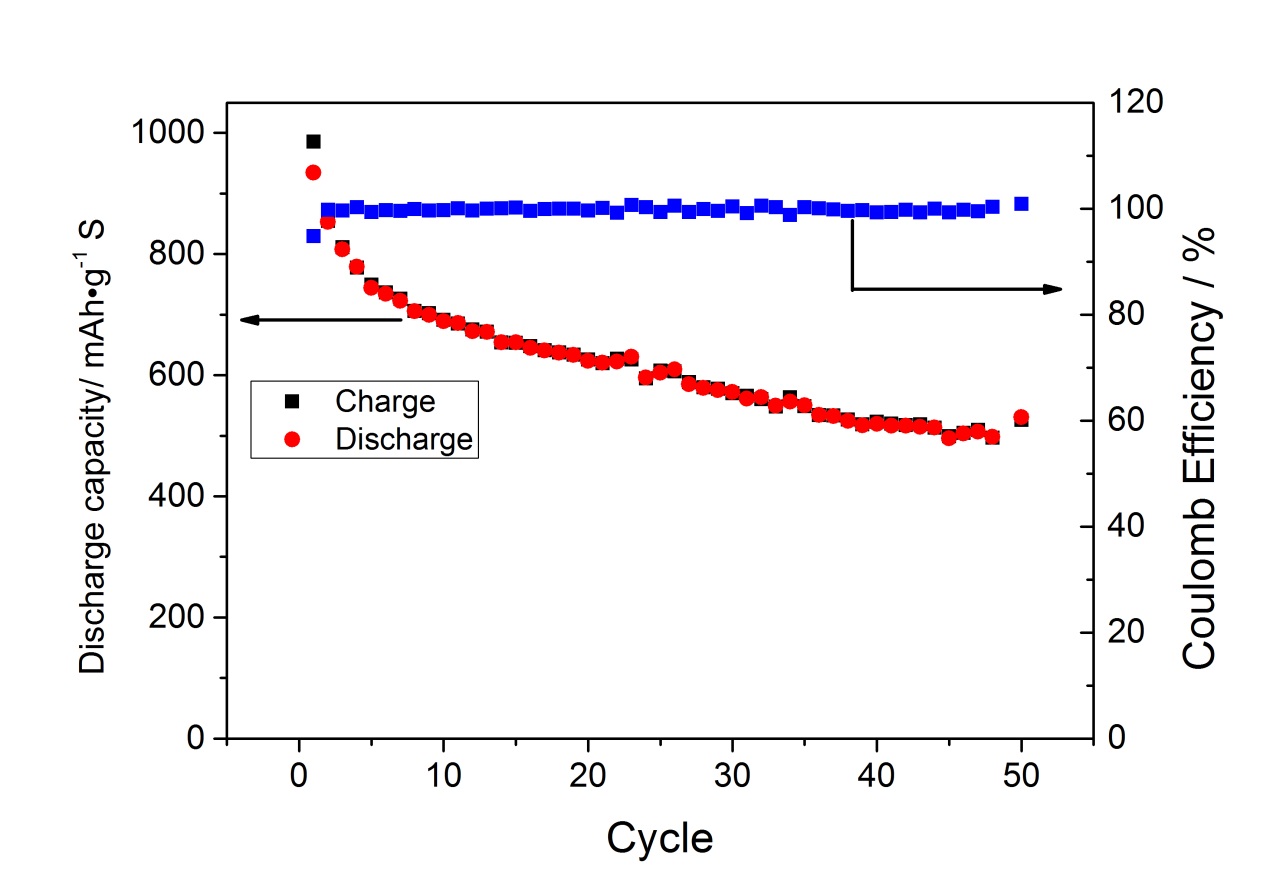


Figure S4. The cycle performance of the cell containing PDTAB film only at 0.5 C.
